# AI-driven discovery in protein science for immunology and infectious disease research

**DOI:** 10.3389/fbinf.2026.1760257

**Published:** 2026-04-13

**Authors:** Mohamed Helmy, Fatma Shafei, Diogo Pellegrina, Lingling Jin, Amr Alhossary, Heather L. Wilson, Tamer Mansour, Kumar Selvarajoo

**Affiliations:** 1 Vaccine and Infectious Diseases Organization (VIDO), University of Saskatchewan, Saskatoon, SK, Canada; 2 Vaccinology and Immunotherapeutics Program, School of Public Health, University of Saskatchewan, Saskatoon, SK, Canada; 3 Department of Computer Science, University of Saskatchewan, Saskatoon, SK, Canada; 4 Department of Computer Science, Idaho State University, Moscow, ID, United States; 5 Bioinformatics Institute (BII), Agency for Science, Technology and Research (A*STAR), Singapore, Singapore; 6 Bioscience Technology Program, Saskatchewan Polytechnic, Saskatoon, SK, Canada; 7 Chemistry Department, Wesleyan University, Middletown, CT, United States; 8 Pulse for Integrated Solutions GmbH, Unterschleißheim, Bavaria, Germany; 9 Department of Veterinary Microbiology, Western College of Veterinary Medicine, University of Saskatchewan, Saskatoon, SK, Canada; 10 Department of Population Health and Reproduction, University of California, Davis, Davis, CA, United States; 11 Department of Clinical Pathology, College of Medicine, Mansoura University, Mansoura, Egypt; 12 Synthetic Biology Translational Research Program, Yong Loo Lin School of Medicine, National University of Singapore (NUS), Singapore, Singapore; 13 Synthetic Biology for Clinical and Technological Innovation (SynCTI), National University of Singapore (NUS), Singapore, Singapore; 14 School of Biological Sciences, Nanyang Technological University (NTU), Singapore, Singapore

**Keywords:** artificial intelligence, biomedical research, disease surveillance, drug discovery, generative AI, immunology, infectious diseases, pandemic preparedness

## Abstract

Artificial Intelligence (AI) is impacting several aspects of modern life with its ability to enhance decision-making, automate complex tasks, and generate human-like content. It is now an indispensable tool in both everyday life and academic inquiry. In particular, the rapid evolution of AI technologies, especially machine learning, deep learning, and natural language processing (NLP), has given rise to large language models (LLMs), which have transformed how we analyze, interpret, and generate text-based, structured data and unstructured data. Among these, Generative AI (GenAI) has become increasingly popular due to its capacity to create content ranging from text and code to protein sequences and molecular structures, all based on patterns found in large training datasets. GenAI tools can assist with literature reviews, writing support, data processing, hypothesis generation, and code or visualization tasks, although outputs require critical oversight to ensure accuracy and relevance. More advanced GenAI applications include the generation of synthetic data and even the design of biological molecules and materials. Within this broader context, the fields of immunology, vaccinology, and infectious diseases research are witnessing a wave of innovation driven by AI. In this review, we explore how these recent advances in GenAI, especially those based on LLMs, are being applied to immunological research, antibody design, vaccine development, infectious diseases research and pandemic preparedness. This review is structured as a scoping review, aiming to map the rapidly evolving applications of GenAI and LLMs in immunology, vaccine development, infectious disease research, and adjacent biomedical fields. Relevant studies were identified through searching PubMed, Google Scholar and preprint archives and included if they introduced, demonstrated, or benchmarked AI-based approaches with clear relevance to immunology and infectious disease, while older preprints without subsequent peer-reviewed publication were excluded. We aim to provide a comprehensive overview of current contributions, emerging tools and models, and future perspectives of GenAI in transforming how we understand and manipulate immune responses and infectious diseases. Therefore, the reported capabilities should be interpreted as indicative of potential rather than definitive performance.

## Introduction

Recent developments in Artificial Intelligence (AI) are changing the landscape of scientific research, with applications that span data analysis and pattern recognition to hypothesis generation and experimental design (Wang et al., 2023). Among the most impacting developments in AI are generative models, especially Generative AI (GenAI), computational models that learn patterns from large datasets and generate new content, which usually built upon large language models (LLMs), computational models trained on massive corpora that learn statistical relationships between tokens, and can be adapted beyond natural language to model biological sequences such as GPT, BERT, and their domain-specific variants (Parasuraman, 2024). These models are trained on massive datasets and had been reported to generate plausible biological sequences, including protein and nucleotide sequences, based on patterns in training data; however, such outputs should be distinguished from experimentally validated biological function, as these models suggest rather than guarantee biologically accurate or functional designs (Díaz-Navarro et al., 2025). This generative capacity makes them particularly suited for applications in biology and medicine, where vast and complex data must be analyzed, interpreted, and repurposed for downstream innovation (Shaban-Nejad et al., 2024). The rapid adoption of GenAI tools by the scientific community reflects their growing utility in automating and accelerating tasks that traditionally require extensive manual effort or experimental cycles.

Generative AI is not a single methodological entity but encompasses several distinct model families with different conceptual foundations and use cases. Transformer-based language models (e.g., GPT-like architectures) learn statistical relationships between tokens in large corpora and are particularly suited for sequence generation, protein design, immune repertoire modeling, and knowledge synthesis (Parasuraman, 2024; Kuan and Farimani, 2024; Elna et al., 2022). Diffusion models generate data through iterative denoising processes, enabling controlled and structure-aware design of molecules, proteins, and antibodies (He et al., 2025; Watson et al., 2023). Graph-based generative approaches, including graph neural networks and related architectures, operate directly on relational structures such as molecular graphs or interaction networks, making them especially useful for modeling protein-protein interfaces, drug-target interactions, and epitope prediction (Wang F. et al., 2025; Kraus et al., 2025). These paradigms differ in how they represent biological information (sequential, continuous spatial, or relational), and therefore are not methodologically interchangeable when applied to similar biological problems.

In the domains of immunology, vaccine development, and infectious disease research, GenAI is demonstrating tangible impact. These fields deal with highly complex, multiscale systems involving genetic, molecular, cellular, clinical and population-level data, making them fertile ground for AI-driven discovery (Iannantuono et al., 2024). The COVID-19 pandemic further underscored the need for rapid and adaptive responses, highlighting limitations in traditional vaccine pipelines and sparking interest in computational strategies for accelerating immunogen and antibody design (Torrington, 2022; Cannataro and Harrison, 2021). Recent advances have enabled GenAI models to contribute directly to problems such as *de novo* antibody design, prediction of immune escape mutations, vaccine antigen selection, and mapping immunogenic landscapes (Mattei et al., 2024; Bravi, 2024). Furthermore, by integrating omics data, structural biology, and evolutionary information, these models can help prioritize vaccine targets and simulate immune responses *in silico* (Gupta et al., 2025; Thadani et al., 2023).

This review surveys the emerging contributions of AI, with a particular focus on those driven by LLMs, to immunology, vaccine development, infectious diseases, and supporting biomedical fields. We discuss how AI approaches, especially generative models, are used in conjunction with structural modeling, multi-omics integration, and high-throughput screening to advance antibody design, predict immune responses, identify vaccine targets, and support pandemic preparedness. We also highlight applications of AI in related domains such as protein structure prediction, sequence annotation, image analysis, single-cell data interpretation, gene editing, and synthetic biology, each of which contributes critical insights and tools to immunological research.

The review is structured as a scoping review designed to survey and map this rapidly evolving landscape. Relevant publications were identified through searching PubMed, Google Scholar and the preprint archives using terms including “Generative AI,” “large language models,” “immunology,” “infectious disease,” “antibody design,” “epitope prediction,” and “vaccine development.” Tools and models were included if they introduced, demonstrated, or benchmarked an AI-based approach with clear relevance to immunology or infectious diseases, while preprints older than 2024 without peer-reviewed publication were excluded. To maximize accessibility and relevance for experimental immunologists, vaccinologists, virologists, and public health researchers, this review is organized around biological and translational application areas rather than detailed comparisons of AI model architectures, with technical distinctions noted only where they directly inform biological interpretation.

Given the rapid pace of development in GenAI and LLM-based methods, many of the tools discussed in this review are recent and have primarily been evaluated within the context of their original publications. Accordingly, descriptions of model capabilities, performance, and accuracy throughout this manuscript reflect what is reported by the original authors in peer-reviewed studies, rather than independently derived or benchmarked results. At present, standardized experimental benchmarks and large-scale comparative evaluations remain limited for many GenAI applications in immunology and infectious disease research. This review therefore adopts a scoping perspective, aiming to map emerging approaches, reported capabilities, and conceptual advances, while acknowledging that rigorous experimental validation and systematic benchmarking will be essential as the field matures. Finally, the review examines current limitations and offers a perspective on future directions, emphasizing the need for biologically grounded, interpretable, and ethically responsible AI systems to fully realize their potential in biomedical research.

## From hypothesis-driven research to AI-driven research

**FIGURE 1:**
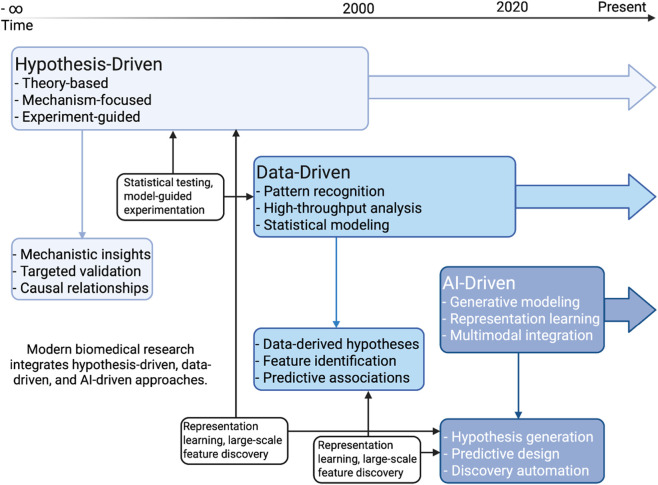
The three scientific approaches and their main properties and outcomes. The white boxes indicate the interaction between approaches and how they are integrated in modern biomedical research.

Scientific research has historically progressed through distinct paradigms of knowledge generation (Figure 1). Hypothesis-driven research, the classical model, is grounded in hypothesis formation and experimental testing based on established theoretical frameworks (Rao, 2019). This approach has yielded foundational advances in immunology and vaccinology, where mechanisms such as clonal selection and immune memory were elucidated through carefully designed experiments and analytical reasoning (Burnet, 1976).

The advent of high-throughput technologies and bioinformatics allowed the promotion of the data-driven research model. In this model, large-scale datasets, such as transcriptomes, proteomes, and pathogen genomes, drive discovery through complex data analytics that include mathematical, statistical, and computational analysis (Fong et al., 2018). This shift enabled pattern recognition, systems-level modeling, and hypothesis generation from data itself, rather than from first principles (Marx, 2013; Sirbu et al., 2023).

Today, we are witnessing the rise of a third paradigm, the AI-driven research, catalyzed by advances in machine learning and, more recently, by LLMs and GenAI. This approach moves beyond analyzing data to the generation of new scientific content. These AI systems can learn from representations of biological sequences, structures, and literature, allowing them to generate novel hypotheses, suggest antibody sequences, antigen/epitope prediction or introduce novel vaccine candidates (Kuan and Farimani, 2024; He et al., 2025; Hie et al., 2021). Unlike traditional models that require explicit human reasoning or predefined statistical frameworks, AI-driven research leverages abstract pattern learning from massive, unstructured data, allowing it to integrate into scientific design.

Importantly, these paradigms are not mutually exclusive (Figure 1). The AI-driven approach complements the hypothesis-driven and the data-driven approaches by expanding the capacity for exploration and accelerating iterations. In immunology and vaccine research, this shift holds the potential to shorten development timelines, uncover unexpected biological insights, and make predictive modeling more adaptive and scalable, marking a new era in biomedicine where advanced computational analyses are integral.

## AI-driven research in immunology, vaccinology and infectious diseases

AI is increasingly contributing to discovery in immunology through its capabilities in data integration, pattern recognition, and predictive modeling (Germain et al., 2024). By leveraging multi-omics datasets, including genomic, proteomic, transcriptomic, and clinical records, AI systems have been reported to support modeling of immune responses, prediction of disease trajectories, and identification of candidate therapeutic targets. Such computational approaches have suggested new ways to study antibody maturation, immune repertoire diversity, and mechanisms of immune escape, although many results remain at the proof-of-concept or computational evaluation stage (Germain et al., 2024; Gururaj et al., 2024). Importantly, these tools do not only catalog patterns but are beginning to provide hypotheses about how immune repertoires adapt under selective pressures, such as infection or vaccination.

In vaccine and immunotherapy development, AI-driven frameworks have been applied to predict B- and T-cell epitopes, analyze viral evolution, and prioritize antigens for candidate vaccines (Olawade et al., 2024). Some platforms, built upon large pathogen genomic repositories such as the Global Initiative on Sharing All Influenza Data (GISAID) (Shu and McCauley, 2017), enable real-time surveillance of viral mutations that could affect immune recognition, thereby informing vaccine redesign and supporting pandemic preparedness (Gururaj et al., 2024). Furthermore, models have been reported to predict patient-specific immune signatures that may guide personalized immunotherapy strategies (Zaslavsky et al., 2025). While these applications remain in varying stages of maturity, they collectively demonstrate how AI, especially generative approaches, can complement experimental immunology and provide a foundation for future integration into vaccine design, epitope prediction, antibody engineering, and disease surveillance. Table 1 Overviews some of the AI-based discovery applications in Immunology, Vaccinology and Infectious Diseases.

**TABLE 1:** Overview of some AI-based discovery applications in immunology, vaccinology and infectious diseases.

Application^a^	Tool	Remarks	Benchmarked	References
Antibody design and vaccine development	AbGPT^$^	Utilizes NLP to target precise antigens	No	Kuan and Farimani (2024)
	EVEscape	Evaluates the viral mutations to predict escape variants before they arise	No	Yaling et al. (2024)
	VenusVaccine^$^	Employs dual-attention deep learning model to accurately predict antigen immunogenicity and guide vaccine target selection	No	Li et al. (2025)
TCR-epitope-MHC binding Prediction	TCRmodel2	Rapidly builds accurate 3D models of TCR-epitope-MHC interactions	Ascunce-París et al. (2025)	Wu et al. (2025c)
	GRIP ^$^	Forecasts TCR-epitope interactions, aiding T-cell-based cancer immunotherapies	No	Papanikolaou et al. (2025)
	tcrLM^$^	Learns from protein sequences to predict the specificity of TCR-epitope binding	No	Fang et al. (2024)
	DapPep^$^	Applies domain-adaptive learning for peptide-agnostic prediction	No	Zheng et al. (2024)
	EpicPred	Utilizes attention-based models to infer phenotypes associated with epitope-binding TCRs	No	Jeon et al. (2025)
	TCR-H	Delivers explainable predictions for unseen TCR-epitope binding datasets	Lu et al. (2025)	T et al. (2024)
Antigen-antibody binding prediction	HelixFold-Multimer^$^	Enhances prediction accuracy of antibody-antigen multimeric structures	No	Gao et al. (2024)
	IgGM^$^	Facilitates *de novo* design of functional antibodies and nanobodies by learning antigen-specific constraints	No	Wang et al. (2025b)
	S^2^ALM	Captures intricate antibody features to improve paratope identification and affinity prediction	No	Yin et al. (2024)
	AntiBinder	Uses hybrid encoding and attention to predict binding interfaces	No	Zhang et al. (2024)
	GraphEPN	Integrates spatial information *via* graph neural networks to improve B-cell epitope prediction	No	Wang et al. (2025a)
	SEMA 2.0	Enables prediction of conformational B-cell epitopes using an AI-supported web tool	No	Ivanisenko et al. (2024)
	MAGE ^$^	Produces target-specific human antibody sequences without templates using a fine-tuned PLM.	No	Wasdin et al. (2025)
	Docking Score ML	Supports virtual screening by improving antigen-antibody affinity scoring	No	Liu et al. (2024a)
Infectious disease surveillance	TagGAN	Enhances automatic labeling of data	No	Nawaz et al. (2026)
	Finite Expression Method	Helps derive epidemiological models directly from observational data	No	Du et al. (2024)

^a^ Performance and capabilities are reported as described in the original publications. The Benchmarked column indicates if a standardized, independent benchmarking and/or experimental validation are available for the tool/model and the reference of the benchmarking study. The $ sign indicates that tool/model is a preprint in the time of writing this review.

### Antibody design and vaccine development

The integration of GenAI into antibody design has opened new directions for immunology and therapeutic development by accelerating the generation, optimization, and evaluation of antibody sequences. Models such as the Antibody Generative Pretrained Transformer (AbGPT) take datasets from 61 individual studies curated from Observatory Antibody Space (OAS) and have been reported to design antibody sequences *de novo*, targeting specific antigens (Kuan and Farimani, 2024). Beyond simple sequence generation, recent frameworks incorporate structural constraints and heavy/light chain pairing compatibility through encoder-decoder architectures (Wang Z. et al., 2024), while probabilistic scoring metrics such as log-likelihood scores allow prioritization of candidate sequences with higher predicted biological plausibility (Uçar et al., 2024a). These tools provide new ways to explore antibody repertoire diversity and maturation, enabling computational hypotheses about how antibodies adapt during affinity maturation or respond to antigenic drift in pathogens. Although most of these applications remain at the computational stage, they represent a shift toward AI-assisted interpretation of immune system adaptability.

AI has also been applied to vaccine development, particularly in predicting antigenic properties and immune escape potential. Tools such as EVEscape analyze the viral mutational landscape to anticipate immune escape variants before they appear in circulating populations, was able to predict 66% of high-frequency mutations in SARS-CoV-2 RBD (Yaling et al., 2024), while models like VenusVaccine employ dual-attention architectures to predict antigen immunogenicity and guide vaccine target selection across diverse pathogens, with 84.5% accuracy for bacteria and 91.4% for viruses (Li et al., 2025). Language models have additionally been used to assess antibody activity against influenza hemagglutinin (HA) protein (Barkan et al., 2025), contributing to pandemic preparedness by suggesting which variants might reduce vaccine effectiveness. These applications illustrate how AI-driven approaches can complement experimental immunology and vaccinology by generating early warning signals of antigenic drift and identifying vaccine targets with higher likelihood of population-level protection.

Together, AI-enabled antibody design and vaccine development provide a foundation for tackling critical immunological challenges. By simulating immune repertoire diversity, modeling pathways of immune escape, and suggesting novel antigen-antibody interactions, these tools help address questions central to vaccinology and infectious disease control. At the same time, it is important to note that many reported successes are still early-stage and require experimental validation before clinical translation.

### TCR-epitope-MHC binding prediction

Accurate prediction of T-cell receptor (TCR)-epitope-MHC binding is fundamental to vaccine development and T-cell-based immunotherapies, as it enables the identification of epitopes capable of eliciting robust and specific immune responses. Conventional approaches, such as NetMHCpan-4.1, utilize machine learning frameworks such as NNAlign_MA to predict peptide binding affinities to MHC molecules (Nielsen et al., 2007; Reynisson et al., 2021). While effective, these models are constrained by the limited number of MHC alleles they support, which poses a significant challenge for vaccine design in genetically diverse, outbred populations, such as swine, where broad MHC coverage is essential. To refine these predictions, molecular docking tools like CABS-Dock, followed by molecular dynamics (MD) simulations and free energy analyses, can be employed to assess the stability and biological plausibility of epitope-MHC complexes (Khatooni et al., 2023; Khatooni et al., 2025). However, these traditional computational workflows are time-consuming, computational expensive, and limited in scalability.

AI-driven approaches offer a powerful alternative by learning directly from large-scale structural and sequence datasets to predict TCR-epitope-MHC interactions with greater speed, accuracy, and generalizability, potentially overcoming the allele coverage and throughput limitations of existing methods. Models such as TCRmodel2 enable rapid and accurate (∼80% with CAPRI Medium accuracy or better) modeling of TCR-epitope-MHC complexes, providing insights into potential TCRs for targeted therapies (Yin et al., 2023). Additionally, generative models, such as generative reconstruction of antigen peptides (GRIP) model, offer predictions for TCR-epitope interactions, supporting advancements in T-cell-based cancer immunotherapies (Papanikolaou et al., 2025). Another generative model, tcrLM, predicts TCR-epitope binding specificity by learning from protein sequences (AUROC of 0.94 on external COVID-19 datasets), thus providing a better understanding of how TCRs interact with peptides, which is vital for advancing T-cell-based cancer immunotherapies (Fang et al., 2024).

Alternatively, deep learning techniques continue to improve TCR-epitope interaction predictions. DapPep employs domain-adaptive learning to make peptide-agnostic predictions (AUROC = 0.82 for unseen peptides), applicable across various TCR-epitope binding scenarios (Zheng et al., 2024). Pretrained language models enhance predictions by incorporating molecular representations and epitope-binding data (Qi et al., 2025; Leary et al., 2024). Models such as EpicPred leverage attention-based learning to predict phenotypes driven by epitope-binding TCRs, improving immune response predictions (average AUROC of 0.80) (Jeon et al., 2025), while TCR-H offers explainable predictions for TCR-epitope binding on unseen datasets, aiding in the interpretation of complex immune data (T et al., 2024). Although these models show promise, most remain in early evaluation stages, with computational predictions awaiting systematic experimental validation. Their potential impact on immunology lies in enabling more comprehensive mapping of immune repertoires, improving the design of T-cell vaccines, and supporting personalized immunotherapies by predicting patient-specific TCR-epitope recognition.

### Antigen-antibody binding prediction

Several AI models have been introduced to predict antigen-antibody interactions, showing potential to support immunotherapy development and diagnostics, though most are in early stages and require further experimental validation. HelixFold-Multimer improves multimeric structure prediction of antibody-antigen complexes, facilitating accurate modeling for therapeutic design (Gao et al., 2024). IgGM is a generative model that enables *de novo* design of functional antibodies and nanobodies by learning antigen-specific constraints (Wang R. et al., 2025). S^2^ALM, a sequence-structure pre-trained LLM, captures complex antibody features for improved paratope and affinity prediction (Yin et al., 2024).

Another group of models, such as AntiBinder, employ hybrid encoding and attention mechanisms to predict binding interfaces (Zhang et al., 2024), and GraphEPN that uses graph neural networks to improve B-cell epitope prediction by integrating spatial features (Wang F. et al., 2025). SEMA 2.0 supports conformational B-cell epitope prediction through an AI-powered web platform (Ivanisenko et al., 2024), while MAGE uses a fine-tuned protein language model (PLM) to generate target-specific human antibody sequences without a template (Wasdin et al., 2025). Additionally, Docking Score ML allows for docking-based virtual screening by refining antigen-antibody affinity assessments (Liu H. et al., 2024). Collectively, these models exemplify how AI accelerates antibody discovery.

### Infectious disease surveillance and pandemic preparedness

Effective infectious disease surveillance is a cornerstone of global health, detecting outbreaks early, mitigating their impact, and preventing future pandemics. AI is improving surveillance methodologies by integrating generative modeling, LLMs, and physics-informed frameworks to enhance data extraction, forecasting precision, and interpretability. Advanced LLMs have demonstrated superior capability in assimilating multifaceted epidemiological datasets, enabling real-time forecasting of pandemic trajectories, as evidenced in SARS-CoV-2 studies (Du et al., 2025). Generative architectures such as TagGAN improve automated data annotation, facilitating scalable and accurate surveillance datasets essential for model training and validation (Nawaz et al., 2026). LLMs have been leveraged for real-time epidemic surveillance by extracting granular epidemiological events and synthesizing heterogeneous data streams, thereby improving situational awareness and decision-making during public health crises (Du et al., 2025; Boussina et al., 2024; Consoli et al., 2024).

Complementing these data-centric approaches, hybrid approaches that combine mechanistic epidemiological models with AI-driven inference offer opportunities for more interpretable and adaptive forecasting. Causal spatiotemporal graph neural networks (Han et al., 2025) and methods like the Finite Expression Method (FEM) (Du et al., 2024), have been reported to capture complex outbreak dynamics and derive governing equations directly from surveillance data. While these systems remain at early stages and require further experimental and field validation, they represent promising directions for building surveillance tools that are not only predictive but also interpretable to public health practitioners. Ultimately, by enhancing the detection of emerging variants, anticipating immune escape, and supporting real-time epidemic forecasting, AI-driven surveillance directly informs vaccine design and infectious disease preparedness.

Beyond outbreak detection and situational awareness, AI plays an increasingly important role in pandemic preparedness by accelerating the development and deployment of medical countermeasures. Generative and predictive AI models can rapidly prioritize therapeutic candidates through drug repurposing and *de novo* design, shortening timelines that are critical during emerging outbreaks. For example, generative molecular design frameworks such as REINVENT and related transformer-based models have been applied to explore large chemical spaces for antiviral candidates (Gawehn et al., 2016; Uçar et al., 2024b), while agentic language models such as TxGemma demonstrate how biomedical knowledge integration can support therapeutic reasoning and prioritization during health emergencies (Umansky et al., 2025). In parallel, AI-driven approaches to protein and antibody modeling enable rapid assessment of antigenic changes, supporting timely vaccine redesign and therapeutic antibody updates as pathogens evolve (Yaling et al., 2024; Kuo et al., 2023). These capabilities are particularly valuable when experimental screening capacity is limited or when rapid decisions must be made under uncertainty.

AI also contributes to pandemic preparedness through the integration of surveillance, evolutionary, and immunological data into adaptive response frameworks. Models that analyze viral genomic variation and immune escape potential, such as EVEscape (Yaling et al., 2024), can help anticipate antigenic drift and guide proactive vaccine updates before escape variants become widespread (Barkan et al., 2025). When combined with real-time epidemiological surveillance and mechanistic or physics-informed models (Boussina et al., 2024; Han et al., 2025; Du et al., 2024; Paul et al., 2021; Zheng et al., 2019), AI systems can inform scenario planning, resource allocation, and intervention strategies at both local and global scales. While many of these applications remain primarily computational and require further experimental and real-world validation, they illustrate how AI-driven tools can complement traditional public health infrastructures by enabling faster hypothesis generation, therapeutic prioritization, and iterative response planning. Continued investment in standardized evaluation frameworks, data sharing, and alignment with public health workflows will be essential for translating these advances into effective pandemic preparedness and response.

### Target therapy and drug discovery

Conventional drug discovery relies on virtual or high-throughput screening and molecular dynamics simulations, which are costly, slow, and often yield limited success (Paul et al., 2021; Zheng et al., 2019). GenAI offers an alternative by generating novel drug-like molecules through architectures such as generative adversarial networks (GANs), variational autoencoders (VAEs), and transformer-based models (Gómez-Bombarelli et al., 2018). Platforms like REINVENT and MolGPT (Olivecrona et al., 2017; Bagal et al., 2022), as well as Atomwise and Insilico Medicine (Gawehn et al., 2016), have been reported to accelerate the exploration of chemical space and improve prediction of drug–target interactions. Early demonstrations include the design of inhibitors against previously “undruggable” targets such as KRAS and SARS-CoV-2 proteins (Zhavoronkov et al., 2019; Huang et al., 2024). While these examples highlight AI’s potential to shorten discovery pipelines, the several outputs remain computational predictions that require extensive experimental validation before clinical application (Chen et al., 2023).

In immunology and infectious disease contexts, GenAI is increasingly applied to therapeutic antibody discovery and refinement. Diffusion-based generative models incorporate structural and evolutionary constraints to propose candidate antibodies (Zhu et al., 2024), while humanization frameworks, methodologies used to convert non-human antibodies into human-compatible antibodies, can adapt non-human antibodies to clinical use without sacrificing binding affinity (Gordon et al., 2024). Additional methods, including retrieval-augmented diffusion models (Wang Z. et al., 2024) and probabilistic scoring schemes (Uçar et al., 2024b), support the prioritization of candidates most likely to succeed experimentally. Although these tools are still at early stages, they point to a growing role for AI in complementing laboratory-based antibody design and in informing therapeutic strategies for infectious diseases Table 2. Overviews some of the AI-based discovery applications in Drug Discovery.

**TABLE 2:** Overview of some AI-based discovery applications in disease-specific modeling and drug discovery.

Application^a^	Tool	Remarks	Benchmarked	References
Target therapy and drug discovery	REINVENT	Uses reinforcement learning to optimize molecular structures for targeted therapies	Yue et al. (2025)	Olivecrona et al. (2017)
	MolGPT	Employs a GPT-based model to generate molecules specific to given targets	Liu et al. (2025)	Bagal et al. (2022)
	TxGemma	Introduces agentic LLMs capable of biomedical reasoning to aid therapeutic decision-making	No	Wang et al. (2025)
	Me-LLaMA	Extends LLM functionality to clinical settings	Diekmann et al. (2024)	Xie et al. (2024)
	RxRx3-core	Benchmarks drug-target interactions using advanced graph learning	No	Kraus et al. (2025)
	GS-DTA	Predicts binding affinity between drugs and targets using both sequence and graph-based models	No	Luo et al. (2025)
Disease-specific AI Models	GALILEO^$^	Expands chemical search space with AI to uncover novel broad-spectrum antiviral compounds	No	Umansky et al. (2025)
	CancerLLM^$^	Enhances prognostic and treatment insights using clinical and biomedical corpora	No	Li et al. (2024)
	Orion	Detects early-se lung cancer with high sensitivity by analyzing circulating oncRNA.	No	Karimzadeh et al. (2024)
	SYN-LUNGS^$^	Enables image analysis by training datasets generation, utilizing anatomically accurate pulmonary nodules DTs	No	Tushar et al. (2025)
	FreeTumor	Synthesizes high-resolution tumor images	No	Wu et al. (2025a)
	ECgMLP	Improves diagnostic classification for endometrial cancer	No	Sheakh et al. (2025)
	GWO + RuleFit	Predicts treatment results in non-small cell lung cancers	No	Duan et al. (2024)
	cancerSimCraft	Models tumor development at single-cell and nucleotide resolution	No	Jin et al. (2024)
	CINner	Simulates chromosomal instability in cancer at the single-cell level	No	Dinh et al. (2025)

^a^ Performance and capabilities are reported as described in the original publications. The Benchmarked column indicates if a standardized, independent benchmarking and/or experimental validation are available for the tool/model and the reference of the benchmarking study. The $ sign indicates that tool/model is a preprint in the time of writing this review.

### Disease-specific AI models

Disease*-specific* models are essential tools in infectious disease research as they offer deeper understanding of pathogen behavior and host responses and help tailor vaccination and treatment strategies to the unique characteristics of each disease. AI is a powerful tool for constructing disease-specific computational models that elucidate pathophysiological mechanisms, enhance diagnostic precision, and optimize therapeutic interventions. The GALILEO generative framework demonstrates the use of AI-driven chemical space expansion for the rapid, one-shot identification of novel antiviral compounds at high hit rates, although these outputs remain computational predictions requiring experimental validation, with reports of activity against Hepatitis C Virus (HCV) and/or human Coronavirus 229E (Umansky et al., 2025). Integrative applications of GenAI with molecular docking and molecular dynamics simulations have identified novel inhibitors targeting critical transcriptional repressors, such as EthR, in *Mycobacterium tuberculosis* (TB), underscoring AI’s role in antimicrobial drug development while still being at the *in silico* proof-of-concept stage (Chikhale et al., 2025). Furthermore, diffusion-guided generative models facilitate the efficient virtual screening of HIV inhibitory molecules, while GANs generate synthetic clinical datasets that address class imbalances found in modeling antiretroviral therapy outcomes (Kuo et al., 2023; Lyu et al., 2024). Together, these studies illustrate the promise of AI in infectious disease-specific modeling, but their biological relevance depends on downstream laboratory confirmation.

In cancer, disease-specific AI models have advanced early detection, prognostication, and personalized therapeutic strategies. The CancerLLM model leverages domain-specific biomedical corpora and clinical datasets to refine prognostic accuracy and therapeutic decision support systems (Li et al., 2024). Orion, a multi-task GenAI model, analyzed circulating orphan non-coding RNAs (oncRNA) and enabled highly sensitive early-stage lung cancer detection, augmenting non-invasive diagnostic methodologies (Karimzadeh et al., 2024). AI-based personalized breast cancer treatment planning systems, informed by the National Comprehensive Cancer Network (NCCN) guidelines, demonstrate the potential for data-driven precision oncology (Mohammed et al., 2025). Multimodal integration of clinical, pathological, radiological, and transcriptomic datasets, coupled with contrastive learning frameworks, advances biomarker discovery and prediction of immunotherapeutic efficacy in metastatic cancers (Arango-Argoty et al., 2025; Captier et al., 2025). While encouraging, these applications are largely context-specific and emphasize the importance of high-quality disease-focused datasets, which are not yet equally available in immunology and infectious disease domains.The SYN-LUNGS platform utilizes anatomically accurate digital twins (DT) of pulmonary nodules, facilitating the generation of training datasets for computed tomography (CT) image analysis and improving model generalizability (Tushar et al., 2025). Complementary to this, FreeTumor generates high-fidelity synthetic tumor images, improving tumor delineation and recognition in radiological workflows (Wu L. et al., 2025). Specialized architectures, such as ECgMLP and GWO + RuleFit, further enable refined diagnostic classification and treatment response prediction in endometrial and non-small cell lung cancers, respectively (Sheakh et al., 2025; Duan et al., 2024). Mechanistic simulation platforms such as cancerSimCraft and CINner enable high-resolution modeling of tumor evolution and chromosomal instability at single-cell granularity, providing indispensable frameworks for benchmarking AI models and exploring intratumoral heterogeneity (Jin et al., 2024; Dinh et al., 2025). Additionally, GenAI facilitates *in silico* synthesis of cancer genomes, addressing data paucity and enhancing the robustness and transferability of predictive models (Díaz-Navarro et al., 2025). These integrative AI methodologies represent a paradigm shift toward mechanistically informed, AI- and data-driven disease modeling driving advances in precision medicine. These examples from oncology highlight the broader trajectory of disease-specific AI models, but translation into immunology and infectious disease research will require comparable investments in domain-specific datasets, mechanistic priors, and rigorous experimental validation Table 3. Overviews some of the AI-based discovery applications in Disease-specific modeling.

**TABLE 3:** Overview of some AI-based discovery applications in relevant biomedical fields.

Application^a^	Tool	Remarks	Benchmarked	References
Protein structure and functional prediction	AlphaFold	Applies transformer and attention-based models to deliver highly accurate protein structure predictions	Xu et al. (2025)	Jumper et al. (2021)
	ESMFold	Predicts protein structures at atomic resolution using a protein language model	Nguyen et al. (2024)	Lin et al. (2023)
	RoseTTAFold	Uses a three-track neural network to predict protein structures and their interactions	Zhai et al. (2025)	Baek et al. (2021)
	ProteinMPNN	Designs protein sequences through deep learning techniques	Li et al. (2025)	Dauparas et al. (2022)
	RFdiffusion	Generates functional protein sequences and structures *de novo*	Wenran et al. (2025)	Watson et al. (2023)
	ProteinBERT	Annotates structural and biophysical attributes, including post-translational modifications, using limited input data	Fang et al. (2024)	Brandes et al. (2022)
	TCR-BERT	deciphers T-cell receptor specificity, predicts B-cell epitopes	Fang et al. (2024)	Wu et al. (2024)
	ESM	Uses evolutionary context for structural and functional protein prediction	Fang et al. (2024)	Lin et al. (2023)
	IgBERT & IgT5	Tailored to manage next-generation antibody sequencing output	Zhang and Tsuda (2025)	Kenlay et al. (2024)
	ProtTrans	Produces universal sequence embeddings for predicting function and subcellular localization	Gao et al. (2025)	Elna et al. (2022)
	PLMSearch	Conducts remote-homology searches using only sequence inputs	Zhang and Freddolino (2024)	Liu et al. (2024b)
	DeepRegFinder	Detects regulatory regions in genomes *via* deep learning	No	Ramakrishnan et al. (2024)
Sequence annotation	DNABERT	Models DNA language to improve prediction of regulatory elements like promoters and TFBSs	Cherednichenko et al. (2025)	Ji et al. (2021)
	DNAHLM^$^	Implements a LLM specifically for DNA analysis	No	Liang (2024)
	VarChat	Connects genomic variants with clinical phenotypes	Gazzo et al. (2025)	De Paoli et al. (2024)
	ChromBPNet	Offers fine-resolution prediction of chromatin accessibility	Avsec et al. (2026)	Pampari et al. (2025)

^a^ Performance and capabilities are reported as described in the original publications. The Benchmarked column indicates if a standardized, independent benchmarking and/or experimental validation are available for the tool/model and the reference of the benchmarking study. The $ sign indicates that tool/model is a preprint in the time of writing this review.

## AI-driven research in supporting biomedical fields

**FIGURE 2:**
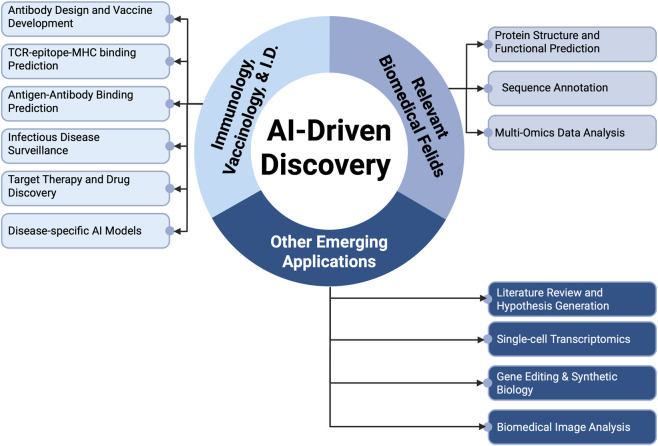
Overview of the contributions of AI-based discovery in immunology, vaccinology, infectious disease research, and relevant fields. *I.D.: infectious diseases.

Advances in immunology and infectious disease research are deeply interconnected with progress in several other biomedical fields, from structural biology and omics to imaging and clinical diagnostics. The recent rise of GenAI and LLMs is not only transforming immunology, vaccinology and infectious disease research directly but also accelerating discovery across several relevant disciplines. GenAI is driving research in protein structure prediction, sequence annotation, multi-omics integration, biomedical image analysis, and clinical decision support, all of which granting more precise and effective research on immune responses and pathogens (Figure 2, Tables 3, 4). In this section, we highlight key applications of GenAI across these complementary areas of biomedical research.

**TABLE 4:** Overview of AI-based applications supporting research in biology and medicine.

Application^a^	Tool	Remarks	Benchmarked	References
Literature review and hypothesis generation	BioBERT	Pretrained on biomedical texts to extract insights and generate answers	Chen et al. (2021)	Lee et al. (2020)
	BioGPT	Trained on millions of biomedical papers to reach near-human performance in research analysis and Q&A	Shang et al. (2025)	Luo et al. (2022)
Single-cell transcriptomics	scVIC	Handles biological variability and batch effects through variational inference	No	Xiong et al. (2024)
	siVAE	Identifies key genes and modules using an interpretable autoencoder, without constructing gene networks directly	No	Choi et al. (2023)
Gene editing and synthetic biology	DNA-Diffusion^$^	Designs regulatory DNA elements in a context-specific manner using diffusion models	No	DaSilva et al. (2024)
	RFdiffusion	Enable *de novo* generation of functional protein sequences and structures	Wenran et al. (2025)	Watson et al. (2023)
	DeepCRISPR	Optimizes CRISPR guided RNA design using deep learning techniques	Konstantakos et al. (2022)	Chuai et al. (2018)
	GuideScan2	Improves precision and effectiveness of CRISPR genome editing	No	Schmidt et al. (2025)
	Evo 2^$^	Enhances large-scale genome design by integrating evolutionary modeling with AI.	Benegas et al. (2025)	Li et al. (2024)
Image analysis	Celldetective	Extracts detailed spatiotemporal features of cells from biomedical images	No	Torro et al. (2025)
	BiomedParse	Provides a unified system for segmenting, detecting, and recognition elements in biomedical imagery	Erdur et al. (2025)	Zhao et al. (2025)
	MediSyn^$^	Generates synthetic medical images based on textual prompts	No	Cho et al. (2024)
	Slideflow	Offers scalable, real-time visualization and analysis of digital pathology whole-slides	No	Dolezal et al. (2024)

^a^ Performance and capabilities are reported as described in the original publications. The Benchmarked column indicates if a standardized, independent benchmarking and/or experimental validation are available for the tool/model and the reference of the benchmarking study. The $ sign indicates that tool/model is a preprint in the time of writing this review.

### Protein structure and functional prediction

Advances in protein structure and functional prediction are foundational to immunology and infectious disease research, as they enable the identification of antigenic determinants, immune recognition interfaces, and molecular mechanisms underlying host–pathogen interactions that directly inform vaccine and immunotherapy design. Accurate structural prediction is critical for mapping epitopes, designing therapeutics, and targeting virulence factors such as invasion proteins, but this remains a complex task due to sequence diversity, folding dynamics, and contextual cellular interactions. Traditional approaches like homology modeling and molecular dynamics were constrained by template dependence and computational cost (Zhang, 2008). The introduction of deep learning–based models such as AlphaFold was a landmark for this research domain, integrating evolutionary, geometric, and implicit physical constraints to achieve near-experimental accuracy in the CASP competition (Jumper et al., 2021; Senior et al., 2020). Other tools, such as RoseTTAFold and ESMFold (Baek et al., 2021; Lin et al., 2023), have extended these advances, while GenAI-driven models like ProteinMPNN and RFdiffusion now enable *de novo* protein design with greater efficiency and diversity than conventional pipelines, as reported by their authors (Watson et al., 2023; Dauparas et al., 2022).

In addition, GenAI-based language models have broadened the scope of protein annotation and immunology-specific predictions. ProteinBERT supports structural annotation and biophysical characterization (Brandes et al., 2022), while derivatives such as TCR-BERT focus on immune receptor specificity and B-cell epitope prediction (Wu et al., 2024). Similarly, models like IgBERT and IgT5 address the complexity of antibody repertoires (Kenlay et al., 2024), ProtTrans provides universal embeddings for functional inference (Elna et al., 2022), and PLMSearch improves homology detection using sequence input alone (Liu W. et al., 2024). AI has also enhanced *de novo* peptide sequencing (π-PrimeNovo, InstaNovo) (Zhang et al., 2025; Eloff et al., 2025), recommended stabilizing mutations (FireProt 2.0) (Musil et al., 2023), and predicted chemical-protein interactions (T5ProtChem) (Kelly et al., 2025). Collectively, these tools offer powerful frameworks for protein analysis and engineering; however, their robustness across immunological contexts, pathogen diversity, and clinical datasets remains under active evaluation.

### Sequence annotation

Accurate sequence annotation underpins immunology and infectious disease research by enabling the identification of immune-relevant genes, antigenic regions, regulatory elements, and functional motifs essential for understanding pathogen biology and host immune responses. GenAI has the potential to accelerate this process by detecting functional DNA, RNA, and protein elements with greater accuracy and minimal manual input. DeepRegFinder, for example, applies deep learning to identify regulatory elements directly from raw data and claimed to outperforming traditional motif-based strategies (Ramakrishnan et al., 2024). Transformer-based models such as BERT-CNN hybrids have been applied to enhancer detection, showing strong performance in recognizing regulatory sequences (Le et al., 2021). Similarly, DNABERT leverages genomic pretraining to improve promoter prediction and transcription factor binding site identification (Ji et al., 2021), while nucleotide-specific models enhance predictions of RNA–protein binding sites (Yamada and Hamada, 2022). In spatial transcriptomics, graph attention networks integrate multi-modal data to define spatial domains, providing richer context for interpreting immune responses in tissues (Huo et al., 2023).

GenAI approaches are also advancing functional interpretation of sequence data. Models like GENERATOR, DNAHLM, and transformer-CNN hybrids refine enhancer and cis-regulatory element detection (Le et al., 2021; Ji et al., 2021; Wu W. et al., 2025; Liang, 2024), while DNA-Diffusion enables *de novo* design of synthetic regulatory elements for controlled gene expression (Ferreira et al.). Tools such as VarChat link genomic variants to clinical outcomes (De Paoli et al., 2024), and ChromBPNet predicts chromatin accessibility at base resolution (Pampari et al., 2025). Collectively, these methods promise to provide high-resolution, context-aware annotations that are especially valuable for understanding immune regulation and host-pathogen interactions. While they represent major progress, many are still in early stages, and their outputs require experimental validation to ensure robustness across diverse biological and clinical settings.

### Multi-omics data analysis

The integration of multi-omics data, spanning genomics, transcriptomics, proteomics, and metabolomics, provides a holistic view of biological systems but presents significant challenges due to data heterogeneity and complexity. GenAI is a powerful tool to tackle those challenges in multi-omics data analysis. For instance, GANs have been applied to multi-omics integration, improving data harmonization and feature extraction while addressing sparsity and batch effects (Ahmed et al., 2021). Similarly, deep learning-based approaches have demonstrated the ability to integrate and analyze complex omics datasets, revealing previously hidden associations between molecular layers and increasing disease classification accuracy (Ballard et al., 2024).

AI-driven models also play a crucial role in handling missing data, a common issue in multi-omics studies. Recent advances in AI have leveraged imputation techniques to reconstruct incomplete datasets, improving the reliability of downstream analyses (Ahmed et al., 2024). Additionally, AI-powered frameworks have been developed to streamline multi-omics workflows, optimizing feature selection and predictive modeling for biomedical research (Flores et al., 2023).

In clinical contexts, AI-powered multi-omics integration has advanced biomarker identification and disease risk prediction. By combining multi-omics with clinical biomarkers, machine learning models have enhanced the power of large cohort studies, such as the UK Biobank, to uncover novel genetic associations and improve disease stratification (Garg et al., 2024). Additionally, AI has improved mass spectrometry-based biomarker discovery, streamlining the detection of clinically relevant proteins and metabolites for precision medicine applications (Tamraparani, 2025). These advancements demonstrate the transformative potential of GenAI in multi-omics data analysis, paving the way for more precise disease diagnostics and efficient vaccination.

### Other emerging applications of AI-driven research

In addition to the above applications, GenAI is driving innovation across a wide spectrum of biomedical domains that provide essential support to immunology and infectious disease research. One important area is literature review and hypothesis generation, where platforms such as BioBERT, PubMedBERT, and BioGPT help in automating the processing and synthesis of scientific knowledge, extraction of novel insights, and identification of emerging research themes (Lee et al., 2020; Gu et al., 2021; Luo et al., 2022). These capabilities accelerate discovery by allowing researchers to navigate vast biomedical corpora more efficiently. Another fast-growing application is in single-cell transcriptomics, where deep generative approaches such as scVIC and siVAE improve clustering, classification, and integration of scRNA-seq with chromatin accessibility data, offering new perspectives on immune heterogeneity, regulatory mechanisms, and host-pathogen interactions (Ding and Regev, 2021; Li et al., 2022). At the same time, AI-driven frameworks show potential in reshaping synthetic biology and genome engineering. Generative models such as DNA-Diffusion and RFdiffusion can design regulatory elements and proteins from scratch, while tools like DeepCRISPR and GuideScan2 help improve the accuracy of CRISPR-based interventions (Watson et al., 2023; DaSilva et al., 2024; Chuai et al., 2018; Schmidt et al., 2025).

In parallel, AI-driven imaging is emerging as a promising tool in biomedical research, though its broader impact is still being established. Recent advances in machine learning have enabled deep learning frameworks to enhance histopathological interpretation, offering more consistent and objective analysis of tissue abnormalities (Kosaraju et al., 2022). Tools such as Celldetective extract spatiotemporal cellular features (Torro et al., 2025), while foundation models such as BiomedParse and specialized pathology AI systems streamline segmentation and improve predictive accuracy for disease classification (Zhao et al., 2025; Wang X. et al., 2024). GenAI further contributes by synthesizing realistic biomedical images to augment training datasets and enable imaging from reduced clinical input, such as reconstructing 3D structures from low-dose X-rays (DeLacey, 2024). Cross-modal tools such as MediSyn enable text-guided image generation (Cho et al., 2024), and platforms such as Slideflow bring scalable, real-time analysis to digital pathology (Dolezal et al., 2024). Collectively, these tools set new standards for biomedical imaging workflows, diagnostic precision, and discovery in immune-related and infectious diseases, though still in their early stages with their outputs requiring careful experimental validation.

## Digital twins: predictive modeling in immunology and infectious disease

Digital twins (DTs) are dynamic, virtual representations of physical systems that continuously integrate data from their real-world counterparts to simulate, monitor, and predict performance in real time (Helmy et al., 2024). Originating from industrial engineering and manufacturing, the DT concept has expanded rapidly into healthcare and biomedical research, where it offers the potential to model complex biological systems at individual or population scales (Bruynseels et al., 2018). By incorporating multi-dimensional data (physiological signals, genomic information, and environmental inputs), DTs can evolve over time to reflect the changing state of the biological entity they represent. This enables continuous, personalized simulations that support diagnostics, treatment planning, and real-time decision-making (Corral-Acero et al., 2020). As digital health technologies advance, DTs are expected to play a more important role in precision medicine by bridging data, computation, and patient care in a continuous feedback loop (Björnsson et al., 2020).

Since DTs are high-fidelity computational replicas of biological systems, they are emerging as powerful platforms for modeling individual or population-level immune responses and pathogen dynamics (Laubenbacher et al., 2024). These virtual models dynamically integrate real-time and historical data streams (e.g., multi-omics, physiological measurements, and environmental exposures) to simulate and predict disease progression, immune function, and treatment outcomes. In immunology and infectious disease research, DTs could enable personalized vaccine design, optimized therapeutic strategies, and *in silico* trials that test intervention efficacy before clinical deployment (e.g., tuberculosis and pneumonia host-pathogen models) (Joslyn et al., 2022; Puniya et al., 2021). By combining mechanistic insights from immunology with patient-specific data, these systems promise to address variability in individual immune profiles, accelerate epidemic response, and support public health.

Despite their promise, the implementation of digital twins for biomedical applications faces significant scientific, technical, and ethical challenges. At the core is the complexity of accurately modeling dynamic and multiscale biological systems as molecular, cellular, physiological, and behavioral processes are deeply intertwined and difficult to represent with precision. Building and maintaining a real-time, data-driven twin requires longitudinal, high-resolution datasets that are often incomplete, noisy, or heterogeneous across patients and contexts (De Domenico et al., 2025). For immunology and infectious diseases, these challenges are even more pronounced due to the stochastic and individualized nature of immune responses, the diversity of pathogens and host-pathogen interactions, and the variability in exposure history and genetic background across populations (Vallée, 2023). Additionally, computational models must be able to evolve over time while maintaining biological plausibility and clinical relevance, which is a nontrivial task given current limitations in mechanistic understanding and model validation. In addition to that, there are several ethical and regulatory concerns, including data privacy, consent, and algorithmic transparency, further complicate adoption, especially when DTs are used for clinical decision support or population-level surveillance (Topol, 2019). As a result, while prototypes and use cases exist, fully functional and generalizable DTs in immunology and infectious diseases remain largely challenging.

**FIGURE 3:**
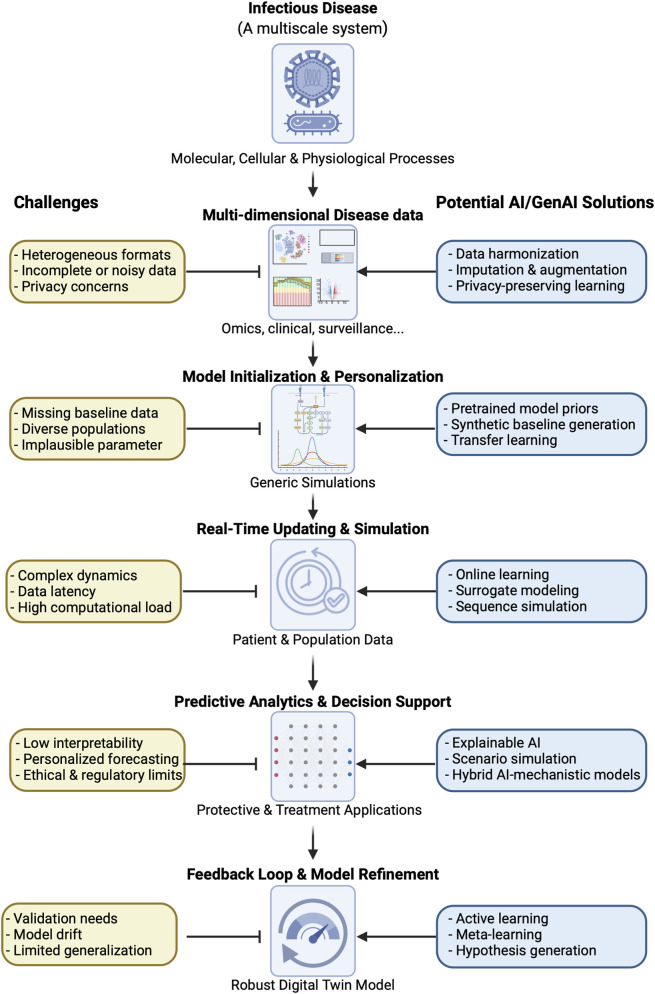
AI-based discovery provides solutions to several challenges in the predictive and personalized *in silico* modeling approaches such as digital twin modeling.

AI, particularly generative models and LLMs, offer promising solutions to many of the challenges faced in developing and deploying DTs in immunology and infectious disease research, though their practical implementation remains in early stages. AI-driven approaches can extract and harmonize heterogeneous data types, including genomics, transcriptomics, imaging, clinical records, and real-time biosensor data, making it feasible to populate and update digital twins with high-dimensional, multimodal information (Liu et al., 2019). GenAI models also can simulate missing or sparse data points, generate plausible patient trajectories, and support *in silico* experimentation by predicting biological responses to interventions or perturbations, such as infection or vaccination. AI has been used to model immune receptor diversity, predict immune responses, and simulate pathogen-host interactions, providing critical components for constructing immune-relevant digital twins (Bruynseels et al., 2018). Moreover, AI systems can enhance the interpretability and scalability of digital twins through dynamic modeling, probabilistic inference, and adaptive learning, allowing the digital counterpart to evolve in real time as new data becomes available. Figure 3 summarizes the challenges facing digital twins modeling and the potential AI solution(s) for each challenge.

## Evaluation and benchmarking of AI models in immunology

Rigorous evaluation and benchmarking are essential for assessing the reliability, generalizability, and translational readiness of AI-driven discovery in immunology, vaccinology, and infectious disease research. While many GenAI and LLM-based tools report strong performance in their original publications, these evaluations are often conducted on curated or domain-specific datasets that may not fully capture biological diversity, population heterogeneity, or rare immune contexts. Independent benchmarking efforts have shown that model performance can vary substantially when applied to less common epitopes, underrepresented MHC alleles, or distribution-shifted datasets. For example, large-scale comparative analyses of 21 TCR-epitope prediction models have demonstrated that while several methods perform reasonably on frequently observed epitopes, most fail to generalize to rarer epitopes, highlighting limitations in robustness and real-world applicability (e.g., comprehensive TCR-epitope benchmarking studies) (Drost et al., 2025). Similarly, community benchmarking initiatives such as IMMREP competitions have emphasized that reported accuracies are highly sensitive to dataset composition and evaluation protocols, underscoring the need for standardized benchmarks and transparent reporting of failure modes (Nielsen et al., 2024).

Independent evaluations further illustrate how reported performance may diverge from outcomes observed under broader or more stringent benchmarking conditions. For example, tFold-TCR was recently published as a high-throughput, end-to-end model for atomic-level prediction of TCR-pMHC complexes, with the authors reporting substantial improvements over AlphaFold-3 based on DockQ prediction success metrics (Wu F. et al., 2025). However, a subsequent independent benchmark comparing general-purpose structure prediction tools (including AlphaFold-3, Boltz-2, and Chai-1) with TCR-specific models (tFold-TCR and TCRmodel2) reported a different performance profile (Ascunce-París et al., 2025). In the benchmark study, tFold-TCR did not consistently achieve high- or medium-quality structural predictions and instead largely reached acceptable-quality predictions on the evaluated test sets, underperforming relative to AlphaFold-3. This example highlights the importance of independent benchmarking across diverse datasets and evaluation criteria, particularly for complex immune recognition problems where dataset composition, epitope diversity, and evaluation thresholds can strongly influence perceived model performance.

These observations illustrate that reported or claimed performance should be interpreted as an indicative of potential rather than definitive capability, particularly in high-dimensional and biologically complex immune systems. Furthermore, these findings reinforce the need for continuous, independent evaluation of AI models using diverse, biologically realistic datasets and experimentally grounded benchmarks. As the field matures, the development of shared standards, reference datasets, and community-driven benchmarking frameworks will be critical to ensure reproducibility, enable fair comparison across methods, and guide responsible integration of AI tools into experimental immunology, vaccine development, and infectious disease research.

## Conclusion and future perspective

Scientific research has undergone a profound transformation over the past few decades, driven not only by technological advances but also by shifting norms in data sharing and accessibility. In earlier years, the culture of open exchange allowed researchers to freely request and receive materials such as plasmids, clones, and sequence data, often with little more than informal attribution or co-authorship. However, as the potential for commercialization increased, intellectual property considerations introduced more formal restrictions through material transfer agreements and institutional policies. More recently, the open science movement, fueled by the mandates of funding agencies and publishers, has supported a culture of transparency. Publications are increasingly expected to be open access, and data are now deposited in public repositories with proper citation frameworks to ensure credit to data generators and their funding sources. This evolving ecosystem of openness and accessibility is a critical enabler for AI-driven research in immunology, vaccinology, and infectious diseases, where large, diverse, and high-quality datasets are essential for robust and interpretable models.

The rapid advancement of GenAI and LLMs has unlocked unprecedented opportunities for discovery across immunology, vaccinology, infectious disease research, and their supporting biomedical disciplines. From predicting protein structure and antigen-antibody interactions to guiding vaccine design and generating therapeutic antibodies, AI-driven research has demonstrated powerful capabilities that are beginning to reshape the scientific process. At the same time, these tools remain early-stage in many applications. Their outputs should be interpreted as computational *predictions* that require careful experimental validation rather than definitive biological conclusions. Systematic benchmarking against experimental gold standards is essential for assessing real-world performance, robustness, and translational readiness. However, such comparative evaluations remain limited due to the rapid emergence of these methods and the lack of standardized, community-wide benchmarking frameworks. Consequently, the capabilities and performance characteristics discussed here reflect what has been reported by the original authors in peer-reviewed publications or computational studies, rather than independently validated consensus outcomes. As the field continues to mature, dedicated benchmarking efforts, integrating experimental validation, reproducibility assessment, and domain-specific performance metrics, will be critical for translating AI-driven advances into reliable biological and clinical applications.

Another key limitation of current GenAI systems is their tendency to generate hallucinated or biologically implausible outputs. These errors are particularly concerning in high-stakes applications such as drug discovery and clinical decision support, where inaccuracies can lead to flawed hypotheses, wasted resources, or harmful clinical consequences. For example, language models may confuse medications with similar names because they rely on approximate pattern matching and can tolerate spelling or wording similarities, even when those drugs have important clinical differences. For instance, Prednisone can be mixed with prednisolone. While both are steroids, Prednisone is a prodrug converted by the liver into the latter. Prescribing the prodrug for patients with inefficient liver functions will render the patient unable to utilize it, leading to possibly serious consequences. Another possible error may include mixing-up drugs with distant functions like Cycloserine (an antibiotic) and Cyclosporine (an immunosuppressive). These risks are amplified in biomedical domains where subtle contextual differences (e.g., tissue-specific expression, variant pathogenicity, or immune receptor specificity) can have major implications. Additionally, many GenAI models are trained on general-purpose corpora or insufficiently annotated datasets, limiting their domain specificity and interpretability. Detailed discussions on limitations and challenges facing GenAI systems in medicine and biology have been extensively explored in the broader literature (Templin et al., 2024; Ennab and Mcheick, 2024; Rouzrokh et al., 2025).

Another challenge lies in the opaque nature of many deep learning models. While attention mechanisms and model explanation tools are improving, the “black box” aspect of many GenAI systems remains a barrier to widespread trust and adoption in scientific and clinical settings. Equally important are the real-world implementation challenges: data scarcity in immunology, limited MHC allele coverage across species, inequities in data access, and model drift in surveillance systems. These practical constraints must be acknowledged when evaluating claims of maturity or generalizability. Regulatory, ethical, and reproducibility concerns further complicate deployment, especially when AI-generated results are used to guide experimentation or therapeutic design. Moreover, equitable access to high-quality training data and computational resources remains unevenly distributed across research institutions and countries, potentially exacerbating existing disparities in global health research.

Another critical challenge for the responsible adoption of AI-driven methods in immunology, infectious disease research, and pandemic preparedness is bias in the underlying data used to train and evaluate these models. Many widely used genomic, immunological, and clinical datasets are disproportionately derived from populations in high-income countries, leading to systematic underrepresentation certain populations and marginalized groups (Bentley et al., 2017; Sirugo et al., 2019). This imbalance risks reducing model accuracy, generalizability, and equity when applied to diverse host genetics, pathogen lineages, and epidemiological contexts. In addition, disparities in access to computational infrastructure, curated datasets, and technical expertise may limit the ability of researchers in resource-constrained settings to develop, validate, or deploy AI-based tools, potentially reinforcing existing global inequities in biomedical research and public health capacity. While AI technologies offer opportunities to democratize analysis through open-source models and cloud-based platforms, without deliberate efforts to diversify training data, support inclusive data sharing, and align AI development with local research priorities, these tools may inadvertently exacerbate disparities rather than alleviate them (World Health Organization, 2021; Schwalbe and Wahl, 2020). Addressing these challenges will require coordinated investment in globally representative data collection, transparent reporting of dataset composition and limitations, and collaborative frameworks that enable equitable participation in AI-driven research and pandemic preparedness.

Looking forward, immunology-specific priorities should guide GenAI applications. These include developing more diverse immunological datasets (e.g., repertoire sequencing across populations), advancing immune escape and epitope diversity modeling, and strengthening benchmarks that directly compare AI predictions with experimental immunology outcomes. Immunologists should also be supported in building AI literacy to critically evaluate outputs and foster meaningful collaborations with computational scientists. Future developments should prioritize the integration of biological priors and mechanistic knowledge into generative frameworks to improve both performance and interpretability. Hybrid approaches that combine empirical learning with systems biology, structural modeling, and domain-specific ontologies will likely yield more robust and biologically grounded outputs. Continued benchmarking of GenAI tools against experimental data, as seen in recent antibody design studies, will also be crucial for validating model outputs and informing model refinement. Experimental validation pipelines, linking AI predictions to wet-lab confirmation, will be critical to ensure biological and clinical relevance.

The field will also benefit from the development of patient-specific and disease-specific AI models that incorporate patient heterogeneity, pathogen diversity, and spatiotemporal dynamics of outbreaks. Digital twin models represent a natural progression in data-driven and AI-driven research, offering the potential to create dynamic, personalized simulations of immune function, disease progression, and treatment outcomes. While their implementations in immunology and infectious diseases are still in development, the integration of genAI, mechanistic modeling, and multimodal data holds great promise for overcoming current limitations in scalability, interpretability, and real-time adaptability. AI-driven tools can help bridge the gap between complex biological processes and clinically actionable models by generating missing data, simulating immune responses, and enabling *in silico* experimentation. However, realizing the full potential of DTs will require continued investment in data infrastructure, model validation, and ethical frameworks, as well as collaborative efforts across disciplines. As digital twin technology matures, it will become a cornerstone of precision medicine and infectious disease preparedness, enhancing how we understand and manage immune-related health challenges.

In tandem, AI models that support synthetic biology and genome engineering, such as those used to optimize CRISPR-Cas targeting or design synthetic regulatory elements, will expand the design space for next-generation vaccines and immunotherapies. In the clinical sphere, multimodal GenAI systems that synthesize omics data, medical imaging, and clinical records hold promise for personalized medicine, early diagnosis, outbreak prevention and real-time epidemic response.

In conclusion, AI is not merely a tool for automating existing workflows; it represents a third wave of biomedical discovery, complementing hypothesis-driven and data-driven approaches. Its impact in immunology will depend on realistic integration: building richer datasets, developing interpretable models, and validating predictions through experiments. By refining, benchmarking, and responsibly deploying GenAI systems, the research community can unlock new levels of speed, precision, and insight, ultimately advancing vaccine development, infectious disease preparedness, and our understanding of immune function.
