# Supplementary material for: AI-driven discovery in protein science for immunology and infectious disease research
Source: Front Bioinform. 2026 Apr 13;6:1760257. doi: 10.3389/fbinf.2026.1760257 (PMC13111274; doi:10.3389/fbinf.2026.1760257)
Supplement: Supplementary file 1 [file DataSheet1.pdf]

Supplementary Tables

Supplementary Table 1. Technical details of some AI-based discovery applications in Immunology, Vaccinology and Infectious Diseases.

Application*	Tool	Utilized Data	Reported Accuracy	Reported Limitations	Ref.
Antibody Design and Vaccine Development	AbGPT$	A dataset drawn from a collection of 61 individual studies curated from Observatory Antibody Space (OAS)	The authors reported the generation of synthetic BCR library of over 15,000 light and heavy chain sequences. Then they used multiple tools (e.g., BioPhi) and visualizations (e.g., t-SNE) to test them.	lower structure prediction scores was observed in certain CDRH3 sequences suggesting it is not suitable yet for all the antibody space	(1)
	EVEscape	Historical viral sequences with structural and biophysical constraints and validated using SARS-Cov-2 sequences from GISAID	Linear regression line shown with a 95% confidence interval. The model predicted 66% of high-frequency observed substitutions during the pandemic.	NA	(2)
	VenusVaccine$	Three curate datasets with immunogenic (positive) and non-immunogenic (negative) labels from bacterial, viral, and human tumor sources	Outperformed baseline methods, achieving highest accuracy and strong discrimination between positive and negative samples.	NA	(3)
TCR-epitope-MHC binding Prediction	TCRmodel2	A custom database containing 52 096 TCR-related sequences and 137 991 MHC-related sequences.	Improved modeling accuracy, achieving 20% high-accuracy near-native predictions and outperforming prior versions and baseline methods.	Limited with the contents of the curated database. Currently focused on modeling the structures of the TCRs and TCR–pMHC complexes.	(4)
	GRIP $	A dataset of 27,231 entries of epitope peptides, CDR beta amino acid sequences and MHCs obtained from publicly available datasets.	The model achieved a training accuracy of 97% and a test accuracy of 85% for predicting epitope sequences at the amino acid level.	The authors reported limitations related to predictions accuracy, data imbalance and lack of integration of structural information.	(5)
	tcrLM$	The model is pretrained ysing a largest TCR CDR3 sequence set with more than 100 million distinct sequences collected from more than ten databases and publications	Surpasses existing TCR-antigen binding prediction methods, but also outperforms other mainstream protein language models	NA	(6)
	DapPep$	Adopted PanPep datasets and control dataset contains 60,333,379 non-binding TCRs (negative samples).	Outperforming existing methods, particularly for unseen peptides like exogenous or neoantigens (PR-AU and AU-ROC).	No real-worked validation. Structural information and gene expression data are not integrated.	(7)
	EpicPred	Six public TCR databases, 244 552 TCR sequences and 105 unique epitope.	An average AUROC of 0.80 ± 0.07	NA	(8)
	TCR-H	TChard dataset consists of 147,069 negative and 107,376 positive datapoints.	AUC of ROC of 0.87, 0.92 and 0.89 for the epitope hard split, TCR hard split and the epitope/TCR strict split.	No quantitatively predict the affinity.	(9)
Antigen - Antibod y Binding Predicti	HelixFold-Multimer$	Extensive dataset of generic protein complexes sourced from the Protein Data Bank (PDB), comprising entries recorded up to September 30, 2021	DockQ score of 0.469 and a success rate of 58.2% overperforming AlphaFold and RoseTTAFold.	Not used in practical antibody development projects	(10)

	IgGM$	A structural dataset using a distillation approach to train a consistency model.	TM-Score: 0.985, DockQ: 0.36	The absence of wet lab experimental data to be used for the validation of the predictions.	(11)
	S^2ALM	Pre-trained over 75 million sequences and 11.7 million structures (UniRef50, PDB, AFDB and OAS databases)	pLDDT: 0.91, pTM: 0.62, ipTM:0.48	NA	(12)
	AntiBinder	Cov-AbDab database containing antibodies and nanobodies targeting coronaviruses with 11 868 studies documenting the neutralizing capabilities of antibodies (35,970 antigen–antibody pairs)	AUC:0.9715, MCC:58.26, Acc:89.35	The model relies on IgFold for antibody structure representation and ESM-2 for antigen embedding, both of which may impose constraints.	(13)
	GraphEPN	SAbDab_1323, SAbDab_665 and Blind_42 Datasets (a total of 2,30 nonredundant protein chains and structures).	AUC:0.829, AUPRC:0.433	The predictive accuracy is still limited by the availability of experimental structural data.	(14)
	SEMA 2.0	PDB database as released on 28 December 2023.	AUC:0.77, MCC:0.229, Senstivity:0.7	NA	(15)
	MAGE $	A training database of 18,507 antibody-antigen sequence pairs curated from literature and existing databases and an original dataset of antigen-specific antibody sequences against diverse viral antigens using LIBRA-seq (linking B cell receptors to antigen-specificity through sequencing).	AUC:0.95 + several in silico and lab validations	The lack of functional data incorporated into data engineering and training	(16)
	Docking Score ML	155 well-known cancer targets were downloaded from the RCSB Protein Databank and data from ChEMBL databases. Also, the DUDE, DUD-AD, and LIT-PCBA as benchmark datasets.	F1 score:84~95, Accuracy:90~100%	Significant discrepancies were observed for certain targets, where the performance gap between the best and worst results exceeded 15%.	(17)
Infectious Disease Surveillance	TagGAN	The Chest eXpert dataset that contains 224,316 chest radiographs drawn from 65,240 distinct disease patients.	NA	Outperform current state-of-the-art methods by approximately 6+% in accuracy	(18)
	Finite Expression Method	The publicly available COVID-19 dataset from Our World in Data and a Synthetic Epidemiological Data created using multiple models.	Successful prediction of actual COVID-19 active cases, deceased cases and recovered cases for 100 days window	High computational cost and providing nonuniqueness of solution.	(19)

* Performance and capabilities are reported as described in the original publications. The $ sign indicates that tools/model is a preprint in the time of writing this review.

Supplementary Table 2. Technical details of some AI-based discovery applications in Disease-specific modeling and Drug Discovery.

Application*	Tool	Utilized Data	Reported Accuracy	Reported Limitations	Ref.
Target Therapy and Drug Discovery	REINVENT	The ZINC 250K dataset that comprises approximately 250K molecules.	AUC top-K	NA	(20)
	MolGPT	Used two benchmark datasets (MOSES and GuacaMol), composed of 1.9M and 1.6M records, respectively.	Validity:0.994, Uniqueness:0.99, Novelty:0.79, Internal Diversity:0.85	NA	(21)
	TxGemma	leverage the Therapeutic Data Commons (TDC) with over 15 million datapoints across various biomedical entities.	Overperformed current methods by 2.4~17.7%	The performance has not yet been validated in real-world, wet-lab experiments.	(22)
	Me-LLaMA	A dataset integrates a vast collection of biomedical literature from PubMed Central and PubMed Abstracts, sourced from the Pile dataset.	outperform ChatGPT on 7 out of 8 datasets and GPT-4 on 5 out of 8 datasets.	Generating information with factual errors or biases. The current token handling capacity, capped at 4096 tokens	(23)
	GS-DTA	Utilized two widely recognized benchmark datasets, Davis and KIBA.	Davis::CI:0.903,MSE:0.21, r2m:0.7; KIBA::CI:0.905,MSE:0.124, r2m:0.806	The fusion of hybrid graph neural networks increases the model complexity. Not incorporating the structural information of proteins which is important for predicting DTA.	(24)
Disease-specific AI Models	GALILEO[$]	Used the ChemPrint dataset, comprises a set of small molecules with well-documented antiviral activity.	In vitro validated, with all 12 NCEs exhibited antiviral activity (100% hit rate).	NA	(25)
	CancerLLM[$]	Two datasets of 2,676,642 cancer clinical notes and 515,524 pathology reports from the University of Minnesota (UMN) Clinical Data Repository	F1 score of 91.78% on phenotyping extraction and 86.81% on disganois generation and outperformed existing LLMs, with an average F1 score improvement of 9.23%.	NA	(26)
	Orion	The TCGA smRNA-seq database to identify 255,393 NSCLC-specific oncRNAs through differential expression analysis of NSCLC and non-cancerous tissues. An in-house dataset of serum collected from 1050 treatment-naive individuals sourced from two different suppliers.	Overall sensitivity of 94% (95% CI: 87%–98%) at 87% (95% CI: 81%–93%) specificity for cancer detection across all stages	NA	(27)
	SYN-LUNGS[$]	The dataset includes 3,072 nodule images from 1,044 simulated CT scans, 512 lesions, and 174 digital twins.	Models trained on clinical + simulated data outperform clinical-only models, with 10% improvement in detection, 2–9% in segmentation, 2–9% in classification and enhanced synthesis.	The unique digital twins remain limited due to full-chest modeling complexity. Also, the simulation pipeline mimics two NLST-era vendor configurations only.	(28)
	FreeTumor	A curate a large-scale dataset comprising 161,310 Computed Tomography (CT) volumes for tumor synthesis and recognition, with only 2.3% containing annotated tumors.	Surpasses the baseline by significant margins, achieving 3.8%~10.6% Dice score improvements.	Limitations in the effectiveness of FID55 in reflecting tumor synthesis quality.	(29)
	ECgMLP	A human curated dataset consists of 3302 images in jpeg format.	Achieves 99.26 % accuracy, surpassing prior diagnostic techniques.	No clinical validation.	(30)
	GWO+RuleFit	Data of 28 patients diagnosed with locally advanced lung cancer.	AUC: 0.58–0.86, p = 0.170–0.925	The method employed for dividing tumor subregions may not capture all relevant features that could potentially enhance the	(31)

				accuracy. The current study is limited by the availability of patient data, which may have affected the generalization.	
	cancerSimCraft	Utilized haplotype copy number profiles from a breast cancer 212 sample that was previously analyzed using CHISEL.	MAE:0.012~0.29, ARI:0.97~1.0	NA	(32)
	CINner	TCGA pan-cancer dataset and the Pan-Cancer Analysis of Whole Genomes (PCAWG).	Undisclosed.	Currently limited to CIN and is not suitable to be use to investigate Chromosomal rearrangements or how the amplifications and deletions of specific alleles might impact the selection landscape differently.	(33)

* Performance and capabilities are reported as described in the original publications. The $ sign indicates that tools/model is a preprint in the time of writing this review.

Supplementary Table 3. Technical details of some AI-based discovery applications in Relevant Biomedical Fields.

Application*	Tool	Utilized Data	Reported Accuracy	Reported Limitations	Ref.
Protein Structure and Functional Prediction	AlphaFold	Proteins from Protein Data Bank and UniClust30	Median backbone accuracy of 0.96 Å RSMD	NA	(34)
	ESMFold	325k structures from PDB, augmented with 12M structures predicted with AlphaFold2.	TM-score of 0.83 on CAMEO and 0.68 on CASP14	NA	(35)
	RoseTTAFold	Protein structures in the Protein Data Bank (PDB)	Average TM-score of 0.8 on CASP14 and CAMEO.	NA	(36)
	ProteinMPNN	19,700 structures from the Protein Data Bank.	Sequence recovery of 52.4% on native protein backbones	NA	(37)
	RFdiffusion	Structures sampled from the Protein Data Bank (PDB)	Median RMSD of 23Å for proteins with 1000 amino acids.	NA	
	ProteinBERT	106M proteins from UniProtKB and UniRef90	74% accuracy on TAPE secondary structure benchmark.	NA	(38)
	TCR-BERT	88,403 TRA and TRB sequences from VDJdb and PIRD	91% AUPRC averaged across 26 antigens	1) Lacks experimental validations. 2) does not leverage VDJ gene usage information or CDR1/2 sequences in its design	(39)
	IgBERT & IgT5	2G sequences from Observed Antibody Space.	Pearson correlation between 0.51 and 0.83 depending on validation dataset.	Focused in antibody specific properties, lacks experimental validation on proposed designs	(40)
	ProtTrans	Annotated protein sequences from Uniref50, UniRef100, and BFD.	Accuracy between 81% and 91% depending on task.	NA	(41)
	PLMSearch	Proteins from SCOPe40-test and Swiss-Prot.	AUROC 0.928 for family-level prediction	NA	(42)
	DeepRegFinder	Annotations from SCREEN and EnhancerAtlas 2.0, further validated with GSE120861.	Validation rates of around 95%	NA	(43)
Sequence Annotation	DNABERT	Promoters annotations from the Eukaryotic Promoter Database.	0.965 accuracy identifying promoter regions	NA	(44)
	DNAHLM$	Human genome sequences and English Wikipedia.	Accuracy around 80% for different tasks.	Ignores other biological characteristics of DNA	(45)
	VarChat	Undisclosed	Undisclosed	NA	(46)
	ChromBPNet	Own ATAC-seq experiment and other ATAC-seq and DNase-seq from ENCODE.	AUROC = 0.98 while discriminating peaks from background.	ChromBPNet doesn't evaluate or predicts predict distal chromatin QTLs.	(47)

* Performance and capabilities are reported as described in the original publications. The $ sign indicates that tools/model is a preprint in the time of writing this review.

Supplementary Table 4. Technical details of some AI-based applications supporting research in biology and medicine.

Application*	Tool	Utilized Data	Reported Accuracy	Reported Limitations	Ref.
Literature Review and Hypothesis Generation	BioBERT	4.5B words over 15 different datasets.	Average F1 score of 0.79 in 3 validations.	NA	(48)
	BioGPT	Trained on millions of biomedical papers to reach near-human performance in research analysis and Q&A.	78% accuracy on PubMedQA.	NA	(49)
Single-cell Transcriptomics	scVIC	5 scRNAseq datasets totaling 92655 cells.	Adjusted Rand Index of ~0.9.	NA	(50)
	siVAE	177,376 cells covering 40 cell types from fetal liver atlas.	0.9 spearman correlation between measured and expected co-expression centrality.	NA	(51)
Gene Editing & Synthetic Biology	DNA-Diffusion$	733 biosamples from 438 cell and tissue types.	Training models showed spearman correlations between 0.45 and 0.77 in 5 test sets.	can't differentiate individual object instances without pixel post processing	(52)
	RFdiffusion	Structures sampled from the Protein Data Bank (PDB).	Median RMSD of 23Å for proteins with 1000 amino acids.	NA	(39)
	DeepCRISPR	1st dataset: ~15,000 sgRNAs containing 1071 genes from four different cell lines. 2nd dataset: 293-related cell lines (18 sgRNAs) and K562 t (12 sgRNAs). 3rd dataset: ChIP-Seq assay, chromatin-opening information from the DNase-Seq assay, and DNA methylation information from the RRBS assay, obtained from ENCODE.	AUC:0.857 while benchmarking sgRNA on-target efficacy prediction.	3 paragraphs of limitations.	(53)
	GuideScan2	CRISPR-based screen of 7 cell lines.	Spearman correlation of 0.44 ($p < 0.001$) between the experimentally defined and GuideScan2-estimated specificities.	NA	(54)
Image Analysis	Celldetective	Human cell lines were cultured and used to generate RICM microscopy data	Lymphocyte detection accuracy of 0.86.	NA	(55)
	BiomedParse	45 publicly available biomedical segmentation datasets across 9 imaging modalities, comprising 1.1 million images, 3.4 million image-mask-label triples, and 6.8 million image-mask-description triples.	BiomedParse outperforms another method of biomedical image segmentation with a p-value of 0.01.	Can't differentiate individual object instances without pixel post processing	(56)
	MediSyn$	1,260,826 publicly available images covering 6 specialties and 10 image types.	When asking a panel of 10 experts to identify the synthetic images from a mixture of real or synthetic ones; the surgeons achieved a recall of 50.59 ± 25.81% and a precision of 63.68 ± 7.39%, while the ophthalmologists achieved a recall of 43.53 ± 17.57% and a precision of 62.39 ± 12.79%.	Trade-off between image quality and privacy of patients in training data. Also, training data is skewed toward radiology.	(57)
	Slideflow	8,122 slides and paired pathologist-annotated regions of interest from The Cancer Genome Atlas.	HPV prediction in slides had a AUROC of 0.81.	Some functions are not cross-compatible between Tensorflow and PyTorch.	(58)

* Performance and capabilities are reported as described in the original publications. The $ sign indicates that tools/model is a preprint in the time of writing this review.

References

1. Kuan D, Farimani AB. AbGPT: De Novo Antibody Design via Generative Language Modeling. (2024) https://arxiv.org/pdf/2409.06090 [Accessed February 20, 2026]

2. Yaling L, Aiping W, Zhou HY. EVEscape: Revealing potential escape sites based on the viral variation landscape. *Biophys Rep* (2024) 10:133. doi: 10.52601/BPR.2024.240902

3. Li S, Tan Y, Ke ⇤ Song, Hong L, Zhou B. IMMUNOGENICITY PREDICTION WITH DUAL ATTEN-TION ENABLES VACCINE TARGET SELECTION. *The Thirteenth International Conference on Learning Representations*. (2025) https://github.com/songleee/VenusVaccine. [Accessed February 16, 2026]

4. Yin R, Ribeiro-Filho H V., Lin V, Gowthaman R, Cheung M, Pierce BG. TCRmodel2: high-resolution modeling of T cell receptor recognition using deep learning. *Nucleic Acids Res* (2023) 51:W569–W576. doi: 10.1093/nar/gkad356

5. Papanikolaou A, Sivtsov V, Zereik E, Ruggiero E, Bonini C, Bonsignorio F. A Simple Generative Model for the Prediction of T-Cell Receptor - Peptide Binding in T-cell Therapy for Cancer. *bioRxiv* (2025)2025.03.18.643937. doi: 10.1101/2025.03.18.643937

6. Fang X, Yu C, Tian S, Liu H. tcrLM: a lightweight protein language model for predicting T cell receptor and epitope binding specificity. (2024) https://arxiv.org/pdf/2406.16995v2 [Accessed February 20, 2026]

7. Zheng J, Xu Q, Xia R, Li SZ. DapPep: Domain Adaptive Peptide-agnostic Learning for Universal T-cell Receptor-antigen Binding Affinity Prediction. (2024) https://arxiv.org/pdf/2411.17798 [Accessed February 20, 2026]

8. Jeon J, Yu S, Lee S, Kim SC, Jo HY, Jung I, Kim K. EpicPred: predicting phenotypes driven by epitope-binding TCRs using attention-based multiple instance learning. *Bioinformatics* (2025) 41: doi: 10.1093/BIOINFORMATICS/BTAF080

9. T RR, Demerdash ONA, Smith JC. TCR-H: explainable machine learning prediction of T-cell receptor epitope binding on unseen datasets. *Front Immunol* (2024) 15:1426173. doi: 10.3389/FIMMU.2024.1426173/BIBTEX

10. Gao J, Hu J, Liu L, Xue Y, Zhu K, Zhang X, Fang X. Precise Antigen-Antibody Structure Predictions Enhance Antibody Development with HelixFold-Multimer. (2024) https://arxiv.org/pdf/2412.09826 [Accessed February 20, 2026]

11. Wang R, Wu F, Gao X, Wu J, Zhao P, Yao J. IgGM: A Generative Model for Functional Antibody and Nanobody Design. *bioRxiv* (2025)2024.09.19.613838. doi: 10.1101/2024.09.19.613838

12. Yin M, Zhou H, Wu J, Zhu Y, Zhan Y, Kong Z, Xu H, Hsieh C-Y, Chen J, Hou T, et al. S2ALM: Sequence-Structure Pre-trained Large Language Model for Comprehensive Antibody Representation Learning. *Research* (2024) doi: 10.34133/research.0721

13. Zhang K, Tao Y, Wang F. AntiBinder: utilizing bidirectional attention and hybrid encoding for precise antibody–antigen interaction prediction. *Brief Bioinform* (2024) 26: doi: 10.1093/BIB/BBAF008

14. Wang F, Dai X, Shen L, Chang S. GraphEPN: A Deep Learning Framework for B-Cell Epitope Prediction Leveraging Graph Neural Networks. *Applied Sciences (Switzerland)* (2025) 15:2159. doi: 10.3390/APP15042159/S1

15. Ivanisenko N V., Shashkova TI, Shevtsov A, Sindeeva M, Umerenkov D, Kardymon O. SEMA 2.0: web-platform for B-cell conformational epitopes prediction using artificial intelligence. *Nucleic Acids Res* (2024) 52:W533–W539. doi: 10.1093/NAR/GKAE386

16. Wasdin PT, Johnson N V., Janke AK, Held S, Marinov TM, Jordaan G, Vandenabeele L, Pantouli F, Gillespie RA, Vukovich MJ, et al. Generation of antigen-specific paired chain antibody sequences using large language models. *bioRxiv* (2025)2024.12.20.629482. doi: 10.1101/2024.12.20.629482

17. Liu H, Hu B, Chen P, Wang X, Wang H, Wang S, Wang J, Lin B, Cheng M. Docking Score ML: Target-Specific Machine Learning Models Improving Docking-Based Virtual Screening in 155 Targets. *J Chem Inf Model* (2024) 64:5413–5426. doi: 10.1021/ACS.JCIM.4C00072/ASSET/IMAGES/LARGE/CI4C00072_0010.JPEG

18. Nawaz M, Nasir B, Zia T, Hussain Z, Moreira C. TagGAN: A Generative Model for Data Tagging. *Comput Biol Med* (2026) 200:111385. https://arxiv.org/pdf/2502.17836 [Accessed February 20, 2026]

19. Du J, Liang S, Wang C. Learning Epidemiological Dynamics via the Finite Expression Method. (2024) https://arxiv.org/pdf/2412.21049 [Accessed February 20, 2026]
20. REINVENT-Transformer: Molecular De Novo Design through Transformer-based Reinforcement Learning. https://arxiv.org/html/2310.05365v5 [Accessed February 20, 2026]
21. Bagal V, Aggarwal R, Vinod PK, Priyakumar UD. MolGPT: Molecular Generation Using a Transformer-Decoder Model. *J Chem Inf Model* (2022) 62:2064–2076. doi: 10.1021/ACS.JCIM.1C00600/ASSET/IMAGES/LARGE/CI1C00600_0012.JPEG
22. Wang E, Schmidgall S, Jaeger PF, Zhang F, Pilgrim R, Matias Y, Barral J, Fleet D, Azizi S. TxGemma: Efficient and Agentic LLMs for Therapeutics. (2025) https://arxiv.org/pdf/2504.06196 [Accessed February 20, 2026]
23. Xie Q, Chen Q, Chen A, Peng C, Hu Y, Lin F, Peng X, Huang J, Zhang J, Keloth V, et al. Me-LLaMA: Foundation Large Language Models for Medical Applications. *Res Sq* (2024)rs.3.rs-4240043. doi: 10.21203/RS.3.RS-4240043/V1
24. Luo J, Zhu Z, Xu Z, Xiao C, Wei J, Shen J. GS-DTA: integrating graph and sequence models for predicting drug-target binding affinity. *BMC Genomics* (2025) 26:105. doi: 10.1186/S12864-025-11234-4
25. Umansky T, Woods V, Russell SM, Garvey DS, Smith DM, Haders D. GALILEO Generatively Expands Chemical Space and Achieves One-Shot Identification of a Library of Novel, Specific, Next Generation Broad-Spectrum Antiviral Compounds at High Hit Rates. *bioRxiv* (2025)2025.01.17.633620. doi: 10.1101/2025.01.17.633620
26. Li M, Zhan Z, Huang J, Yeung J, Ding K, Blaes A, Johnson S, Liu H, Xu H, Zhang R. CancerLLM: A Large Language Model in Cancer Domain. (2024) https://arxiv.org/pdf/2406.10459 [Accessed February 20, 2026]
27. Karimzadeh M, Momen-Roknabadi A, Cavazos TB, Fang Y, Chen NC, Multhaup M, Yen J, Ku J, Wang J, Zhao X, et al. Deep generative AI models analyzing circulating orphan non-coding RNAs enable detection of early-stage lung cancer. *Nature Communications* (2024) 15:1–12. doi: 10.1038/S41467-024-53851-9;SUBJMETA=114,1305,1350,1612,4028,631,67,692;KWRD=MACHINE+LEARNING,NON-SMALL-CELL+LUNG+CANCER
28. Tushar FI, Dahal L, McCabe C, Ho FC, Segars P, Abadi E, Lafata KJ, Samei E, Lo JY. SYN-LUNGS: Towards Simulating Lung Nodules with Anatomy-Informed Digital Twins for AI Training. (2025) https://arxiv.org/pdf/2502.21187 [Accessed February 20, 2026]
29. Wu L, Zhuang J, Zhou Y, He S, Ma J, Luo L, Wang X, Ni X, Zhong X, Wu M, et al. Large-scale generative tumor synthesis in computed tomography images for improving tumor recognition. *Nature Communications 2025 16:1* (2025) 16:11053-. doi: 10.1038/s41467-025-66071-6
30. Sheakh MA, Azam S, Tahosin MS, Karim A, Montaha S, Fahim KU, Shafiabady N, Jonkman M, De Boer F. ECgMLP: A novel gated MLP model for enhanced endometrial cancer diagnosis. *Computer Methods and Programs in Biomedicine Update* (2025) 7:100181. doi: 10.1016/J.CMPBUP.2025.100181
31. Duan C, Liu Q, Wang J, Tong Q, Bai F, Han J, Wang S, Hippe DS, Zeng J, Bowen SR. GWO+RuleFit: rule-based explainable machine-learning combined with heuristics to predict mid-treatment FDG PET response to chemoradiation for locally advanced non-small cell lung cancer. *Phys Med Biol* (2024) 69:155018. doi: 10.1088/1361-6560/AD6118
32. Jin H, Navin N, Chen K. cancerSimCraft: A Multi-resolution Cancer Genome Simulator with Comprehensive Ground Truth Tracking. *bioRxiv* (2024)2024.12.11.627708. doi: 10.1101/2024.12.11.627708
33. Dinh KN, Vázquez-García I, Chan A, Malhotra R, Weiner A, McPherson AW, Tavaré S. CINner: Modeling and simulation of chromosomal instability in cancer at single-cell resolution. *PLoS Comput Biol* (2025) 21:e1012902. doi: 10.1371/JOURNAL.PCBI.1012902
34. Jumper J, Evans R, Pritzel A, Green T, Figurnov M, Ronneberger O, Tunyasuvunakool K, Bates R, Žídek A, Potapenko A, et al. Highly accurate protein structure prediction with AlphaFold. *Nature 2021 596:7873* (2021) 596:583–589. doi: 10.1038/s41586-021-03819-2
35. Lin Z, Akin H, Rao R, Hie B, Zhu Z, Lu W, Smetanin N, Verkuil R, Kabeli O, Shmueli Y, et al. Evolutionary-scale prediction of atomic-level protein structure with a language model. *Science (1979)* (2023) 379:1123–1130. doi: 10.1126/science.ade2574

36. Nguyen PT, Harris BJ, Mateos DL, González AH, Murray AM, Yarov-Yarovoy V. Structural modeling of ion channels using AlphaFold2, RoseTTAFold2, and ESMFold. *Channels* (2024) 18: doi: 10.1080/19336950.2024.2325032
37. Dauparas J, Anishchenko I, Bennett N, Bai H, Ragotte RJ, Milles LF, Wicky BIM, Courbet A, de Haas RJ, Bethel N, et al. Robust deep learning–based protein sequence design using ProteinMPNN. *Science (1979)* (2022) 378:49–56. doi: 10.1126/SCIENCE.ADD2187/SUPPL_FILE/SCIENCE.ADD2187_SM.PDF
38. Brandes N, Ofer D, Peleg Y, Rappoport N, Linial M. ProteinBERT: a universal deep-learning model of protein sequence and function. *Bioinformatics* (2022) 38:2102–2110. doi: 10.1093/BIOINFORMATICS/BTAC020
39. Watson JL, Juergens D, Bennett NR, Trippe BL, Yim J, Eisenach HE, Ahern W, Borst AJ, Ragotte RJ, Milles LF, et al. De novo design of protein structure and function with RFdiffusion. *Nature 2023 620:7976* (2023) 620:1089–1100. doi: 10.1038/s41586-023-06415-8
40. Kenlay H, Dreyer FA, Kovaltsuk A, Miketa D, Pires D, Deane CM. Large scale paired antibody language models. *PLoS Comput Biol* (2024) 20:e1012646. doi: 10.1371/JOURNAL.PCBI.1012646
41. Elnaggar A, Heinzinger M, Dallago C, Rehawi G, Wang Y, Jones L, Gibbs T, Feher T, Angerer C, Steinegger M, et al. ProtTrans: Toward Understanding the Language of Life Through Self-Supervised Learning. *IEEE Trans Pattern Anal Mach Intell* (2022) 44:7112–7127. doi: 10.1109/TPAMI.2021.3095381
42. Liu W, Wang Z, You R, Xie C, Wei H, Xiong Y, Yang J, Zhu S. PLMSearch: Protein language model powers accurate and fast sequence search for remote homology. *Nature Communications 2024 15:1* (2024) 15:1–12. doi: 10.1038/s41467-024-46808-5
43. Ramakrishnan A, Wangensteen G, Kim S, Nestler EJ, Shen L. DeepRegFinder: deep learning-based regulatory elements finder. *Bioinformatics Advances* (2024) 4: doi: 10.1093/BIOADV/VBAE007
44. Ji Y, Zhou Z, Liu H, Davuluri R V. DNABERT: pre-trained Bidirectional Encoder Representations from Transformers model for DNA-language in genome. *Bioinformatics* (2021) 37:2112–2120. doi: 10.1093/BIOINFORMATICS/BTAB083
45. Kelly T, Xia S, Lu J, Zhang Y. DNAHLM -- DNA sequence and Human Language mixed large language Model. *Cite This: J Chem Inf Model* (2024) 65:3998. doi: 10.1021/acs.jcim.5c00051
46. De Paoli F, Berardelli S, Limongelli I, Rizzo E, Zucca S. VarChat: the generative AI assistant for the interpretation of human genomic variations. *Bioinformatics* (2024) 40: doi: 10.1093/BIOINFORMATICS/BTAE183
47. Pampari A, Shcherbina A, Kvon EZ, Kosicki M, Nair S, Kundu S, Kathiria AS, Risca VI, Kuningas K, Alasoo K, et al. ChromBPNet: bias factorized, base-resolution deep learning models of chromatin accessibility reveal cis-regulatory sequence syntax, transcription factor footprints and regulatory variants. *bioRxiv* (2025)2024.12.25.630221. doi: 10.1101/2024.12.25.630221
48. Lee J, Yoon W, Kim S, Kim D, Kim S, So CH, Kang J. BioBERT: a pre-trained biomedical language representation model for biomedical text mining. *Bioinformatics* (2020) 36:1234–1240. doi: 10.1093/BIOINFORMATICS/BTZ682
49. Luo R, Sun L, Xia Y, Qin T, Zhang S, Poon H, Liu TY. BioGPT: generative pre-trained transformer for biomedical text generation and mining. *Brief Bioinform* (2022) 23:1–11. doi: 10.1093/BIB/BBAC409
50. Xiong J, Gong F, Ma L, Wan L. scVIC: deep generative modeling of heterogeneity for scRNA-seq data. *Bioinformatics Advances* (2024) 4: doi: 10.1093/BIOADV/VBAE086
51. Choi Y, Li R, Quon G. siVAE: interpretable deep generative models for single-cell transcriptomes. *Genome Biol* (2023) 24: doi: 10.1186/S13059-023-02850-Y/TABLES/1
52. DaSilva LF, Senan S, Patel ZM, Reddy AJ, Gabbita S, Nussbaum Z, Córdova CMV, Wenteler A, Weber N, Tunjic TM, et al. DNA-Diffusion: Leveraging Generative Models for Controlling Chromatin Accessibility and Gene Expression via Synthetic Regulatory Elements. *bioRxiv* (2024)2024.02.01.578352. doi: 10.1101/2024.02.01.578352
53. Chuai G, Ma H, Yan J, Chen M, Hong N, Xue D, Zhou C, Zhu C, Chen K, Duan B, et al. DeepCRISPR: Optimized CRISPR guide RNA design by deep learning. *Genome Biol* (2018) 19:1–18. doi: 10.1186/S13059-018-1459-4/FIGURES/7

54. Schmidt H, Zhang M, Chakarov D, Bansal V, Mourelatos H, Sánchez-Rivera FJ, Lowe SW, Ventura A, Leslie CS, Pritykin Y. Genome-wide CRISPR guide RNA design and specificity analysis with GuideScan2. *Genome Biol* (2025) 26:1–25. doi: 10.1186/S13059-025-03488-8/FIGURES/5
55. Torro R, Díaz-Bello B, Arawi D El, Dervanova K, Ammer L, Dupuy F, Chames P, Sengupta K, Limozin L. Celldetective: an AI-enhanced image analysis tool for unraveling dynamic cell interactions. *Elife* (2025) 14:2024.03.15.585250. doi: 10.1101/2024.03.15.585250
56. Zhao T, Gu Y, Yang J, Usuyama N, Lee HH, Kiblawi S, Naumann T, Gao J, Crabtree A, Abel J, et al. A foundation model for joint segmentation, detection and recognition of biomedical objects across nine modalities. *Nat Methods* (2025) 22:166–176. doi: 10.1038/S41592-024-02499-W;SUBJMETA=114,1305,1564,631;KWRD=IMAGE+PROCESSING,MACHINE+LEARNING
57. Björnsson B, Borrebaeck C, Elander N, Gasslander T, Gawel DR, Gustafsson M, Jörnsten R, Lee EJ, Li X, Lilja S, et al. MediSyn: A Generalist Text-Guided Latent Diffusion Model For Diverse Medical Image Synthesis. (2024) doi: 10.1186/s13073-019-0701-3
58. Dolezal JM, Kochanny S, Dyer E, Ramesh S, Srisuwananukorn A, Sacco M, Howard FM, Li A, Mohan P, Pearson AT. Slideflow: deep learning for digital histopathology with real-time whole-slide visualization. *BMC Bioinformatics* (2024) 25:1–29. doi: 10.1186/S12859-024-05758-X/FIGURES/2
